# The Hospital Frailty Risk Score as a Predictor of Mortality, Complications, and Resource Utilization in Heart Failure: Implications for Managing Critically Ill Patients

**DOI:** 10.3390/biomedicines13030760

**Published:** 2025-03-20

**Authors:** Nahush Bansal, Eun Seo Kwak, Abdel-Rhman Mohamed, Vaishnavi Aradhyula, Mohanad Qwaider, Alborz Sherafati, Ragheb Assaly, Ehab Eltahawy

**Affiliations:** 1 Department of Medicine, University of Toledo, Toledo, OH 43606, USA; 2 Division of Pulmonary and Critical Care Medicine, University of Toledo, Toledo, OH 43606, USA; 3 Division of Cardiovascular Medicine, University of Toledo, Toledo, OH 43606, USA

**Keywords:** frailty, heart failure, hospital frailty risk score (HFRS), in-hospital mortality, risk stratification, national inpatient sample (NIS)

## Abstract

**Background:** Frailty, with a high prevalence of 40–80% in heart failure, may have a significant bearing on outcomes in patients. This study utilizes the Hospital Frailty Risk Score (HFRS), a validated tool derived from the administrative International Classification of Diseases, 10th Revision, Clinical Modifications (ICD-10-CM) codes, in investigating the mortality, morbidity, and healthcare resource utilization among heart failure hospitalizations using the Nationwide Inpatient Sample (NIS). **Methods:** A retrospective analysis of the 2021 NIS database was assessed to identify adult patients hospitalized with heart failure. These patients were stratified by the HFRS into three groups: low frailty (LF: <5), intermediate frailty (IF: 5–15), and high frailty (HF: >15). The outcomes analyzed included inpatient mortality, length of stay (LOS), hospitalization charges, and complications including cardiogenic shock, cardiac arrest, acute kidney injury, and acute respiratory failure. These outcomes were adjusted for age, race, gender, the Charlson comorbidity score, hospital location, region, and teaching status. Multivariate logistic and linear regression analyses were used to assess the association between frailty and clinical outcomes. STATA/MP 18.0 was used for statistical analysis. **Results:** Among 1,198,988 heart failure admissions, 47.5% patients were in the LF group, whereas the IF and HF groups had 51.1% and 1.4% patients, respectively. Compared to the LF group, the IF group showed a 4-fold higher (adjusted OR = 4.60, *p* < 0.01), and the HF group had an 11-fold higher (adjusted OR 10.90, *p* < 0.01) mortality. Frail patients were more likely to have a longer length of stay (4.24 days, 7.18 days, and 12.1 days in the LF, IF, and HF groups) and higher hospitalization charges (USD 49,081, USD 84,472, and USD 129,516 in the LF, IF, and HF groups). Complications were also noticed to be significantly (*p* < 0.01) higher with increasing frailty from the LF to HF groups. These included cardiogenic shock (1.65% vs. 4.78% vs. 6.82%), cardiac arrest (0.37% vs. 1.61% vs. 3.16%), acute kidney injury (19.2% vs. 54.9% vs. 74.6%), and acute respiratory failure (29.6% vs. 51.2% vs. 60.3%). **Conclusions:** This study demonstrates the application of HFRS in a national dataset as a predictor of outcome and resource utilization measures in heart failure admissions. Stratifying patients based on HFRS can help in holistic assessment, aid prognostication, and guide targeted interventions in heart failure.

## 1. Introduction

Frailty is defined as a clinically recognizable state of increased vulnerability resulting from an age-associated decline in function across multiple physiologic systems [1]. It plays a pivotal factor in shaping outcomes among hospitalized patients with heart failure. As a syndrome characterized by decreased physiological reserves and vulnerability to stressors, frailty has a profound impact on heart failure outcomes [2]. Affecting 40–80% of patients with heart failure, frailty is particularly prevalent in those with preserved ejection fraction (HFpEF), in whom up to 90% of patient are affected due to the advanced age and increased comorbidity burden in this group [3]. Although less common in HF with reduced ejection fraction (HFrEF), frailty still impacts 30–60% of patients and is increasingly recognized as a distinct biological syndrome that causes physical and cognitive impairments, irrespective of age or other conditions [4,5].

Frailty in heart failure is associated with a 1.5- to 2-fold increase in the risk of all-cause mortality and hospitalizations, making it a critical prognostic marker that may surpass traditional cardiovascular risk factors in significance [4,6]. Research has demonstrated a bidirectional relationship between heart failure and frailty, wherein each condition exacerbates the other [3,7]. The hallmark features of frailty, such as sarcopenia, cognitive decline, and functional impairment, can independently predispose individuals to heart failure and worsen its prognosis [8]. This intersection between heart failure and frailty is rooted in shared pathophysiological mechanisms, including systemic inflammation, neurohormonal dysregulation, and metabolic derangements. Given this intricate interplay and its profound impact on clinical trajectories, a holistic approach that integrates the assessment of frailty into heart failure care is imperative.

Traditionally, frailty in heart failure patients has been assessed using various tools and scales that incorporate physical signs, symptoms, cognitive assessments, and functional tests. However, these methods often lack a comprehensive evaluation of all frailty domains and are less practical in routine clinical settings. The Hospital Frailty Risk Score (HFRS) addresses these limitations by providing a validated, practical tool to identify frailty in hospitalized patients using routinely collected administrative data. The HFRS is derived from 109 International Classification of Diseases, 10th Revision (ICD-10) codes, encompassing frailty-related diagnoses and patterns of healthcare utilization [9]. This approach leverages the globally standardized ICD-10 coding system to capture a wide range of diagnoses, including volume depletion, cognitive impairment, and pulmonary and cardiovascular conditions. The HFRS has been successfully applied across various countries and healthcare systems, highlighting its versatility and applicability in diverse patient populations [10,11].

This study evaluates the impact of frailty, as measured using the HFRS, on in-hospital outcomes including mortality, resource utilization, and complication rates in the patients admitted with heart failure. This study also aims to validate the utility of the HFRS in predicting outcomes for the patients admitted with heart failure, facilitating risk stratification and guiding management strategies.

## 2. Methods

This study is a retrospective cohort analysis of all the adult patients admitted to acute care hospitals in the United States during the year 2021. This study was conducted by using the National Inpatient Sample (NIS), a database developed by the Agency for Healthcare Research and Quality (AHRQ). The NIS is the largest publicly accessible all-payer inpatient database, representing all non-federal acute care hospitals across the country. It employs a stratified sampling design based on hospital characteristics such as ownership, bed size, control, teaching status, geographic region, and urban or rural designation. A 20% probability sample of hospitals is drawn from each stratum, and discharge data are weighted to produce national estimates. The 2021 NIS dataset includes information from 49 statewide organizations, covering 98% of the U.S. population. It provides detailed patient- and hospital-level data, enabling a comprehensive analysis of healthcare utilization, outcomes, and trends.

Patients were identified using the International Classification of Diseases, Tenth Revision, Clinical Modification (ICD-10-CM) coding system. The principal diagnosis codes for heart failure (I50.xx, I09.81, I11.0, I13.0, and I13.2) were used to select all adult patients admitted with heart failure. Frailty was assessed using the Hospital Frailty Risk Score (HFRS), a validated ICD-10 coding algorithm developed by Gilbert et al. and subsequently utilized in various studies [9,10,11]. The HFRS is calculated from a comprehensive list of 109 ICD-10 codes, each assigned a weight ranging from 0.1 to 7.1, reflecting the strength of its association with frailty. The summed scores yield a final frailty risk score, stratified into three cohorts: low frailty (HFRS < 5), intermediate frailty (HFRS 5–15), and high frailty (HFRS > 15). By convention, the intermediate and high frailty cohorts were grouped to represent the frail population to compare baseline characteristics [9].

**Figure 1:**
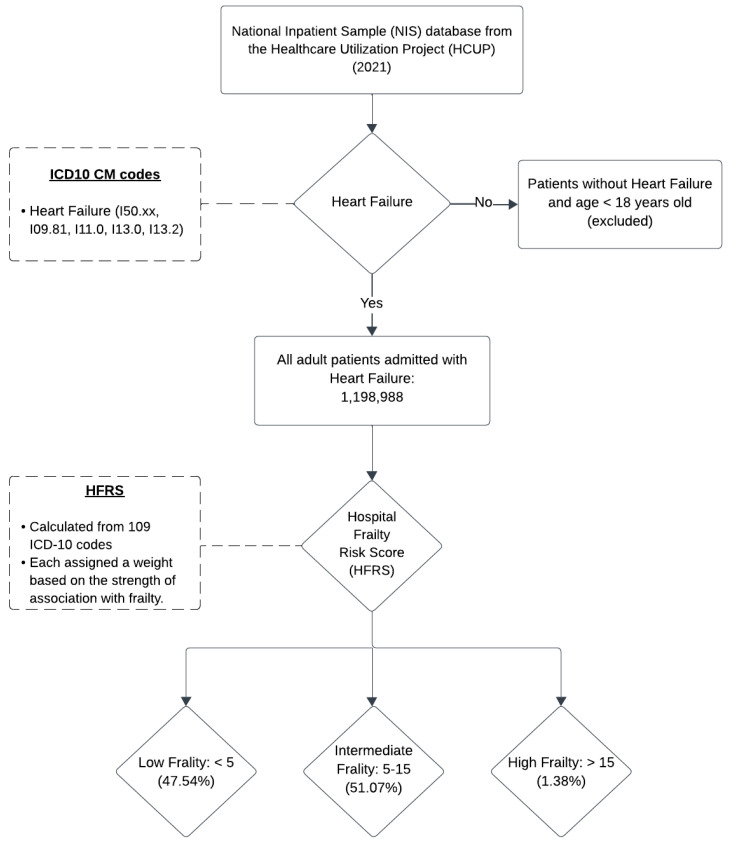
Inclusion and stratification criteria used for heart failure and frailty.

The primary outcome of this study was in-hospital mortality. Secondary outcomes included length of stay (LOS), total hospitalization charges, and the incidence of complications such as cardiogenic shock, cardiac arrest, acute kidney injury, and acute respiratory failure. Supplementary Table S1 provides a comprehensive list of ICD-10 codes used for patient selection. Figure 1 illustrates the patient selection and classification process used in this study.

Data analysis was conducted using STATA/MP version 18.0 (Stata Corp., College Station, TX, USA). Baseline characteristics were compared between frail and non-frail patients with heart failure. A univariate analysis was initially performed to assess various outcomes among heart failure patients. Multivariate logistic regression was subsequently employed to adjust for potential confounders, including age, sex, race, median household income, patient comorbidities (measured using the Charlson Comorbidity Index [CCI]), geographic region (Northeast, Midwest, West, or South), hospital location (rural or urban), teaching status, bed size, and primary payer or insurance status. Continuous variables were expressed as means with 95% confidence intervals (CIs), and regression analysis was utilized to evaluate differences across the frailty subgroups within the heart failure cohort. Categorical variables were compared using the Chi-squared test. A two-sided *p*-value of <0.05 was considered statistically significant throughout the analysis.

The logistic regression model fit was evaluated using the Hosmer–Lemeshow test, which yielded a *p*-value of 0.62, indicating an acceptable fit. Multicollinearity among independent variables was assessed using the Variance Inflation Factor (VIF). All predictors had VIF values below 10, confirming that multicollinearity was not a significant concern. These findings support the robustness of our logistic regression model in evaluating frailty and in-hospital outcomes in heart failure patients. The dataset was examined for missing values in key variables. The proportion of missing data was minimal (<5% across all variables). Given the low level of missing data, no imputation techniques were necessary. A post hoc power analysis confirmed that the study had sufficient statistical power (>99%) to detect clinically meaningful differences in in-hospital mortality and other outcomes, given the large sample size.

## 3. Results

### 3.1. Patient Characteristics

**Figure 2:**
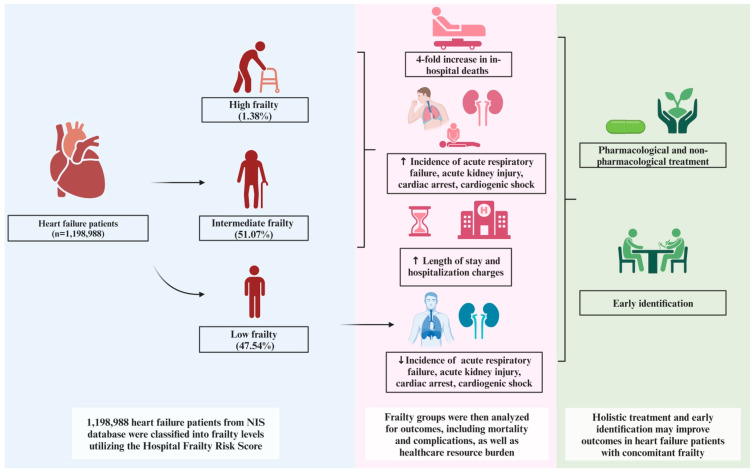
Impact of frailty on various in-hospital outcomes in patients admitted with heart failure.

This study utilized the 2021 NIS database, comprising 1,198,988 patients with a primary diagnosis of heart failure. Among these, 47.54% were classified in the low frailty (LF) subgroup, 51.07% in the intermediate frailty (IF) subgroup, and 1.38% in the high frailty (HF) subgroup. The intermediate and high frailty subgroups collectively constituted the frail population. Frail patients were significantly older (mean age: 72.49 years; *p* < 0.001) compared to non-frail patients (mean age: 68.78 years). Additionally, frail patients were more likely to be female, have a higher Charlson Comorbidity Index (CCI) score, and be insured through Medicare. Table 1 provides a detailed comparison of baseline demographic and hospital-related characteristics between frail and non-frail heart failure patients. Figure 2 illustrates the outcomes observed across the different frailty subgroups in heart failure.

### 3.2. Primary Outcome: Mortality

The overall in-hospital mortality rate for patients admitted with heart failure in 2021 was 2.93%, corresponding to 35,089 patients. Mortality rates significantly increased with rising frailty scores, highlighting a progressive relationship between frailty and death. The high frailty (HF) subgroup exhibited the highest mortality rate at 10.42%, followed by the intermediate frailty (IF) subgroup at 4.68%, and the low frailty (LF) subgroup at 1.06%. Adjusted analyses revealed significantly higher odds of in-hospital mortality for patients in the HF subgroup (adjusted odds ratio [aOR], 9.31; 95% confidence interval [CI], 8.13–10.67; *p* < 0.001) and in the IF subgroup (aOR, 4.26; 95% CI, 3.98–4.56; *p* < 0.001) compared to the LF subgroup (Table 2).

### 3.3. Secondary Outcomes

#### 3.3.1. Resource Utilization: Length of Stay and Hospital Charges

Resource utilization was assessed by analyzing the length of stay (LOS) and hospital charges among heart failure patients. The mean LOS was significantly higher in the high frailty (HF) subgroup at 12.1 days (95% CI: 11.55–12.65; *p* < 0.001), compared to 7.19 days (95% CI: 7.09–7.28; *p* < 0.001) in the intermediate frailty (IF) subgroup and 4.24 days (95% CI: 4.19–4.29) in the low frailty (LF) subgroup. Hospital charges followed a similar trend, with frail patients incurring significantly higher costs. The mean hospital charges for the HF subgroup were USD 129,516.1 (95% CI: 116,874–142,158.2; *p* < 0.01), compared to USD 84,472.95 (95% CI: 81,105.96–87,839.94; *p* < 0.01) in the IF subgroup and USD 49,081.45 (95% CI: 47,667.06–50,495.84; *p* < 0.01) in the LF subgroup. These findings highlight the substantial resource burden associated with frailty in heart failure. Table 2 provides an illustration of these results.

#### 3.3.2. Complications

After adjusting for patient- and hospital-level confounders, patients in the high frailty (HF) and intermediate frailty (IF) subgroups exhibited significantly higher rates of complications compared to the low frailty (LF) subgroup. The rates of cardiogenic shock were notably elevated in the HF subgroup (aOR, 6.43; 95% CI: 5.43–7.61; *p* < 0.001) and the IF subgroup (aOR, 3.59; 95% CI: 3.33–3.86; *p* < 0.001). Similarly, cardiac arrest was significantly more common in the HF subgroup (aOR, 9.54; 95% CI: 7.51–12.11; *p* < 0.001) and the IF subgroup (aOR, 4.48; 95% CI: 3.98–5.03; *p* < 0.001). Other complications followed a similar pattern, with acute kidney injury being markedly more frequent in the HF (aOR, 12.02; 95% CI: 11.03–13.1; *p* < 0.001) and IF subgroups (aOR, 4.86; 95% CI: 4.75–4.96; *p* < 0.001). Additionally, the rates of acute respiratory failure were significantly higher in both the HF subgroup (aOR, 3.66; 95% CI: 3.38–3.97; *p* < 0.001) and the IF subgroup (aOR, 2.58; 95% CI: 2.53–2.64; *p* < 0.001). Table 2 and Table 3 demonstrate these results.

#### 3.3.3. Predictors of In-Hospital Mortality

In a multivariate analysis, factors independently associated with increased in-hospital mortality in patients admitted with heart failure were older age, male sex, a higher Charlson Comorbidity Index, lower quartile income, teaching hospital status and a larger hospital bed size. Table 4 illustrates these findings.

## 4. Discussion

This nationwide study assessing the utility of the Hospital Frailty Risk Score (HFRS) as a predictor of adverse outcomes in patients admitted with acute heart failure revealed several important findings. Frailty was highly prevalent among heart failure patients, with over half of the cohort classified as having intermediate or high frailty. Frail patients demonstrated significantly higher mortality rates, with a more than 4-fold increase in in-hospital deaths among those with higher frailty indices. Additionally, frailty was associated with markedly higher resource utilization, including prolonged hospital stays and increased hospitalization charges. Frail patients also experienced a significantly higher incidence of complications, such as cardiogenic shock, acute respiratory failure, cardiac arrest, and acute kidney injury. Notably, the adverse clinical outcomes observed were proportional to the degree of frailty, with patients in the high frailty subgroup experiencing the most severe outcomes.

In our cohort, 51% of heart failure patients were classified as frail. Globally, the prevalence of frailty in heart failure has been reported to vary widely, ranging from 15% to 80% [12,13,14]. This variation is largely attributable to differences in frailty definitions, measurement tools, study populations, sample sizes, and inclusion criteria across studies. A meta-analysis of various studies estimated the overall prevalence of frailty in heart failure at 44.5% [15], closely aligning with the prevalence identified using the HFRS in our study.

Frailty was associated with more than a 4-fold increase in mortality among patients admitted with heart failure in our cohort. This contrasts with the findings of a pooled study by Yang et al., which reported a 1.5-fold increase in mortality among heart failure patients with frailty [6]. The discrepancy may stem from differences in follow-up duration, patient populations, and study settings. While Yang et al. assessed one-year mortality in patients with chronic heart failure, our study focused on the impact of frailty on inpatient mortality during acute heart failure admissions. Collectively, these findings highlight that the impact of frailty on heart failure outcomes becomes more pronounced during acute exacerbations. Frailty, characterized by diminished physiological reserves, makes individuals more vulnerable to acute illnesses compared to chronic conditions. Post hoc analyses from the GUIDE-IT trial further underscored the adverse impact of frailty on outcomes in patients with HFrEF [16]. Among 879 participants, 56.3% of patients with high frailty experienced significantly worse outcomes, including higher rates of all-cause mortality (20.8% vs. 5.5%) and heart failure hospitalizations (27.6% vs. 21.5%).

The worse prognosis observed in frail patients with heart failure can be attributed to a complex interplay of molecular and cellular mechanisms that exacerbate both conditions. These mechanisms include an enhanced proinflammatory state, neurohormonal activation, immune cell dysregulation, insulin resistance, micronutrient deficiencies, and tissue dysfunction [8]. Chronic inflammation, a hallmark of both frailty and heart failure, is characterized by elevated levels of proinflammatory cytokines such as interleukin-6 and C-reactive protein, which contribute to muscle wasting and functional decline [3,17]. In addition, hemodynamic abnormalities in heart failure, such as chronic congestion and reduced perfusion, further amplify the systemic effects of frailty [10,11,18]. Neurohormonal changes, including increased sympathetic activity and the activation of the renin–angiotensin–aldosterone system, intensify the deterioration of both cardiac and skeletal muscle function. Among micronutrient deficiencies, vitamin D deficiency plays a prominent role in heart failure and is closely linked to frailty. Loop diuretics, commonly used in heart failure management, can exacerbate vitamin D deficiency by increasing urinary excretion [19]. Other micronutrient deficiencies, including selenium, zinc, and vitamin C are also associated with poorer outcomes in heart failure. These physiological impairments contribute to the increased vulnerability of frail patients to stressors associated with heart failure. Importantly, the negative impact of frailty on heart failure outcomes has been shown to occur independently of the severity of cardiac dysfunction [6]. Furthermore, frailty is associated with higher complication rates and prolonged recovery following advanced heart failure interventions, such as left ventricular assist device (LVAD) implantation [13,14].

The timely and accurate diagnosis of frailty in heart failure is critical given its significant impact on outcomes. Various methods and instruments have been employed to assess frailty in heart failure patients. These include identifying frail phenotypes based on predefined criteria, using self-reported questionnaires, and evaluating performance on physical tests such as gait speed, the timed up-and-go test, and hand grip strength [3,17]. However, a systematic review by McDonagh et al. highlighted inconsistencies in the current methods for frailty assessment in heart failure [18]. The Hospital Frailty Risk Score (HFRS), derived from administrative data using standardized ICD-10 codes, has demonstrated robust external validation in predicting clinical outcomes across different countries [10,11]. Our study demonstrates the successful application of the HFRS in the U.S. heart failure population, further validating its utility. The HFRS offers healthcare systems a low-cost, efficient, and systematic approach to screening for frailty. It identifies patients at a higher risk of adverse outcomes, enabling targeted management strategies to improve clinical results.

A holistic treatment approach, incorporating both pharmacological and non-pharmacological strategies, is essential to optimize outcomes in heart failure patients with concurrent frailty. Research indicates that frail heart failure patients are less likely to receive guideline-directed medical therapy (GDMT), contributing to their poorer outcomes [16,20]. A retrospective study of 477 ambulatory HFrEF patients revealed that frail individuals were less likely to be prescribed triple therapy (ACE inhibitors/ARBs, MRAs, and beta-blockers) compared to non-frail counterparts (39% vs. 56%). Moreover, even when prescribed, frail patients were frequently on suboptimal doses [21]. This suggests that physicians may hesitate to initiate or up-titrate GDMT in frail patients, despite evidence indicating that this high-risk group derives amplified benefits from optimal pharmacotherapy. Further research is warranted to identify the barriers limiting GDMT use in frail patients and to develop strategies that address these factors to improve outcomes. Non-pharmacological interventions, such as exercise training and nutritional support, also play a critical role in improving outcomes. A comprehensive, tailored multidomain rehabilitation program aimed at enhancing balance and strength has been shown to improve quality of life and reduce all-cause hospitalization rates in heart failure patients [22]. Nutritional interventions for frail heart failure patients should emphasize a balanced diet, the replacement of specific micronutrients such as vitamin D and iron, and adequate calorie and protein supplementation to enhance muscle mass and tissue function. These targeted interventions have demonstrated success in both outpatient chronic and inpatient acute heart failure settings.

This study has many strengths. First, it leverages a large, nationally representative dataset, ensuring the broad generalizability of the findings to diverse patient populations. Second, by adjusting for multiple patient- and hospital-level confounders, this study provides unbiased and robust estimates of the association between frailty and clinical outcomes. Additionally, our study examines a wide range of inpatient complications, including cardiogenic shock, acute kidney injury, respiratory failure, and cardiac arrest, which have not been comprehensively evaluated in prior studies on frailty in heart failure. These findings provide deeper insights into frailty’s impact on acute care outcomes. Finally, this study demonstrates the practical utility and application of HFRS in predicting in-hospital mortality, complications, and resource utilization in the US healthcare system, reinforcing its potential role in risk stratification and guiding targeted interventions for frail heart failure patients.

This study has several limitations. As the derivation of the HFRS relies on ICD-10 codes, it is susceptible to misclassification bias. Additionally, the National Inpatient Sample (NIS) is an administrative database that lacks detailed clinical information, such as the severity or New York Heart Association (NYHA) classification of heart failure. To partially address this limitation, the Charlson Comorbidity Index, a validated prognostic scale, was utilized [23]. Furthermore, the NIS does not provide key clinical and laboratory parameters, including echocardiographic findings, heart failure etiology, brain natriuretic peptide (BNP) levels, or details on treatments administered to heart failure patients. Despite these limitations, the NIS offers a nationally representative, large-scale dataset with robust administrative data, providing sufficient statistical power to draw meaningful conclusions regarding the impact of frailty and the utilization of the HFRS in heart failure. This study underscores the potential of the HFRS as a valuable tool for predicting outcomes and resource utilization in this population.

## 5. Conclusions

Frailty has a significant impact on in-hospital outcomes for patients admitted with heart failure, including a 4-fold increased risk of mortality and increased burden on healthcare resources, as evidenced by prolonged hospital stays and elevated hospitalization charges. Frailty also amplifies the risk of both cardiac and non-cardiac complications, such as cardiac arrest, cardiogenic shock, acute kidney injury, and acute respiratory failure. These adverse outcomes are directly proportional to the degree of frailty, with higher frailty scores correlating with worse outcomes compared to patients with intermediate frailty. The Hospital Frailty Risk Score (HFRS), a practical tool derived from ICD-10 codes, provides an efficient and reliable means of estimating frailty, enabling effective risk stratification and outcome prediction in heart failure patients. The early identification and targeted management of frailty hold the potential to improve outcomes in this high-risk population. Given these findings, further research is warranted to explore the impact of targeted interventions, such as optimized pharmacological and non-pharmacological strategies, on specific outcomes in heart failure patients with concomitant frailty.

## Figures and Tables

**Table 1:** Demographic and hospital-related characteristics of heart failure patients with and without frailty.

	Frail	Non-Frail	*p* Value
Total (%) of heart failure admissions	52.45	47.55	
Age (Median)	72.49	68.78	<0.01
Female gender (%)	49.72	43.75	<0.01
Race			<0.01
Caucasian	65.92	63.03	
African American	20.23	23.12	
Hispanic	8.76	8.9	
Asian or Pacific Islander	2.38	2.17	
Native American	0.53	0.62	
Others	2.17	2.17	
Median income in patient’s zip code (%)			<0.01
USD 1–47,999	76.72	66.22	
USD 48,000–60,999	10.9	15.29	
USD 61,000–81,999	10.24	14.18	
≥USD 82,000	2.14	4.31	
Charlson Comorbidity Index (%)			0.12
1	4.43	13.85	
2	9.18	20.92	
3 or more	86.39	65.23	
Hospital region			<0.01
Northeast	18.21	18.58	
Midwest	24.39	21.33	
South	40.18	42.54	
West	17.22	17.56	
Hospital bed size (%)			<0.01
Small	23.81	25.62	
Medium	28.48	28.84	
Large	47.71	45.54	
Hospital location (%)			<0.01
Rural	9.07	10.53	
Urban	90.93	89.47	
Hospital teaching (%)			<0.01
Non-teaching (%)	27.33	29.64	
Teaching (%)	72.67	70.36	
Insurance type (%)			<0.01
Medicaid	76.72	66.22	
Medicare	10.9	15.29	
Private	10.24	14.18	
Uninsured	2.14	4.31	

**Table 2:** Unadjusted primary and secondary outcomes stratified by frailty in heart failure admissions.

Variable	Low Frailty	Intermediate Frailty	High Frailty	*p* Value
Deaths (%)	1.06	4.68	10.42	<0.01
Complications (%)				
Cardiogenic shock	2.91	3.55	2.82	<0.01
Cardiac arrest	0.73	0.89	1	<0.01
Acute kidney injury	35.31	38.65	37.75	<0.01
Acute respiratory failure	39.06	30.5	33.9	<0.01
Resource Use				
LOS, d	4.24 (4.19–4.29)	7.19 (7.09–7.28)	12.1 (11.55–12.65)	<0.01
Hospital cost, USD	49,081 (47,667–50,495)	84,472 (81,105–87,839)	129,516 (116,874–142,158)	<0.01

**Table 3:** Adjusted outcomes with confidence intervals among various racial groups in patients with heart failure.

Outcomes (Intermediate vs. Low Frailty)	aOR; 95% CI	*p* Value
Mortality	4.26 (3.98–4.56)	<0.01
Cardiogenic Shock	3.59 (3.33–3.86)	<0.01
Cardiac Arrest	4.48 (3.98–5.03)	<0.01
Acute Kidney Injury	4.86 (4.75–4.97)	<0.01
Acute Respiratory Failure	2.58 (2.53–2.64)	<0.01
Outcomes (High vs. Low Frailty)		
Mortality	9.32 (8.14–10.67)	<0.01
Cardiogenic Shock	6.43 (5.43–7.61)	<0.01
Cardiac Arrest	9.54 (7.51–12.11)	<0.01
Acute Kidney Injury	12.02 (11.03–13.09)	<0.01
Acute Respiratory Failure	3.67 (3.39–3.97)	<0.01

**Table 4:** Multivariate regression analysis with adjusted OR for in-hospital mortality.

Variable	Adjusted Odds Ratio (aOR)	*p* Value
Age	1.02 (1.01–1.03)	<0.01
Female sex	0.88 (0.83–0.93)	<0.01
Charlson Comorbidity Index	1.02 (1.01–1.04)	<0.01
Income Quartile		
USD 1–47,999	Reference	
USD 48,000–60,999	1.11 (1.04–1.18)	<0.01
USD 61,000–81,999	1.02 (0.95–1.09)	0.65
≥USD 82,000	0.98 (0.91–1.07)	0.70
Hospital Teaching Status		
Non-Teaching	Reference	
Teaching	1.12 (1.04–1.22)	<0.01
Hospital Bed Size		
Small	Reference	
Medium	1.02 (0.94–1.11)	0.64
Large	1.18 (1.09–1.28)	<0.01

## Data Availability

The original contributions presented in this study are included in the article/supplementary material. Further inquiries can be directed to the corresponding author.

## Supplementary Materials

The following supporting information can be downloaded at: https://www.mdpi.com/article/10.3390/biomedicines13030760/s1, Table S1: ICD 10-CM codes used for various conditions in the study.
