# Supplementary material for: The Hospital Frailty Risk Score as a Predictor of Mortality, Complications, and Resource Utilization in Heart Failure: Implications for Managing Critically Ill Patients
Source: Biomedicines. 2025 Mar 20;13(3):760. doi: 10.3390/biomedicines13030760 (PMC11940347; doi:10.3390/biomedicines13030760)
Supplement: Supplementary file 1 [file biomedicines-13-00760-s001.zip › biomedicines-3487962-supplementary.pdf]

Table S1. ICD 10-CM codes used for various conditions in the study.

**Condition**	**ICD10-CM Codes**
Heart Failure	I50xx, I0981, I110, I130, I132
Cardiac arrest	I462, I468, I469
Cardiogenic shock	R570
Acute Kidney Injury	N170, N171, N172, N178, N179
Acute Respiratory Failure	J9600, J9601, J9602, J9620, J9621, J9622, J9690, J9691, J9692
